# Obstetric Outcome After Surgical Treatment of Endometriosis: A Review of the Literature

**DOI:** 10.3389/frph.2021.750750

**Published:** 2021-12-24

**Authors:** Samantha S. Mooney, Vanessa Ross, Catharyn Stern, Peter A. W. Rogers, Martin Healey

**Affiliations:** ^1^Department of Gynaecology (Endosurgery), Mercy Hospital for Women, Heidelberg, VIC, Australia; ^2^Department of Obstetrics and Gynaecology, Western Health (Joan Kirner Women's and Children's), St Albans, VIC, Australia; ^3^Department of Obstetrics and Gynaecology, University of Melbourne, Parkville, VIC, Australia; ^4^Department of Gynaecology, The Royal Women's Hospital, Parkville, VIC, Australia

**Keywords:** endometriosis, surgery, laparoscopy, pregnancy, adverse pregnancy (birth) outcomes

## Abstract

A diagnosis of endometriosis is associated with increased risks of adverse pregnancy outcomes including placenta praevia and preterm birth. Some studies have also suggested associations with gestational hypertension, foetal growth restriction, gestational diabetes, perinatal death, and obstetric haemorrhage. This review aims to assess the impact of pre-pregnancy surgical treatment of endometriosis on future obstetric outcomes. A search of the Medline, Embase and PubMed electronic databases was performed to identify studies reporting pre-pregnancy surgery for endometriosis and subsequent pregnancy outcome compared to controls with unresected endometriosis. Three studies met the inclusion criteria. The studies were heterogenous in design, definition of study groups and outcome measures. All three studies were judged at critical risk of bias. Pre-pregnancy excision of endometriosis was associated with an increased risk of caesarean section in one of two studies, OR 1.72 (95% CI 1.59–1.86) and OR 1.79 (95% CI 0.69–4.64). Placenta praevia rates were also increased in one of two studies OR 2.83 (95% CI 0.56–12.31) and OR 2.04 (95% CI 1.66–2.52). One study found increased risks of preterm birth, small for gestational age, gestational hypertension, and antepartum and postpartum haemorrhage (all *p* < 0.05) with pre-pregnancy excision of endometriosis. There is insufficient evidence examining the role of pre-pregnancy endometriosis surgery in ameliorating adverse pregnancy outcomes, and thus reliable conclusions cannot be drawn. Prospectively designed studies are needed to assess the relationship between surgical treatments for endometriosis and obstetric outcome and examine potential confounders such as comorbid adenomyosis and infertility.

## Introduction

Endometriosis is defined as the presence of endometrial-like glands and stroma outside the uterine cavity (1). This oestrogen-dependent chronic condition affects 11.4% of reproductive age women (2) and is associated with pelvic pain and infertility (1).

In recent years, there has been an increasing focus on the association between endometriosis and pregnancy outcomes (3–6). Endometriosis may be associated with poor pregnancy outcomes (7, 8) including placenta praevia, preterm birth (PTB), premature prelabour rupture of membranes (PPROM), obstetric haemorrhage, gestational hypertensive disorders, foetal growth restriction (IUGR) and perinatal death (4). These associations are biologically plausible due to several factors: the inflammatory mileu and immune modifications established by endometriosis (9–11), the molecular, anatomical, and epigenetic abnormalities of eutopic endometrium noted in women with endometriosis (12–14), and the decidualisation of endometriosis lesions due to the hormonal changes of pregnancy (15). Several authors have attempted to demonstrate a causal relationship between endometriosis and obstetric complications (3). To date, studies are yet to explore the possibility of a common pathophysiology which predisposes to both endometriosis and adverse pregnancy outcomes. In addition, no specific management for endometriosis has been proposed to improve adverse obstetric outcomes. It is unknown whether pre-pregnancy surgery for endometriosis alters the risk of adverse obstetric outcomes or if there is a surgically untreatable factor influencing these outcomes. Moreover, the impact of assisted reproductive technologies (ART) on obstetric risk, as well as the influence of comorbid adenomyosis need exploration.

This review aims to summarise the evidence examining the association between adverse obstetric outcomes beyond 20 weeks' gestation and pre-pregnancy surgery for endometriosis.

## Methods

### Search

A search of the literature published between January 2015 and June 2021 was performed in the Medline, Embase and PubMed databases. Articles were restricted to the English language and full text articles. The search included the following keywords and medical subject headings: “endometriosis” and/or “endometrioma” combined with “colorectal surgery” or “general surgery” or “gynaecology” or “urology” or “ablation” or “excision” and further combined with “pregnancy outcome” expanded with “pregnancy complications” which included “preterm birth,” “gestational diabetes,” “gestational hypertension,” “pre-eclampsia,” “antepartum haemorrhage,” “postpartum haemorrhage,” “caesarean section,” “placental abruption,” “intrauterine growth restriction,” “stillbirth,” “placenta praevia,” and/or “cholestasis.”

### Study Selection

Studies were included if they: (i) were prospective or retrospective cohort or case–control studies, (ii) reported on pregnancies beyond 20-weeks gestational age, and (iii) indicated surgical treatment of endometriosis prior to pregnancy. Studies needed to evaluate at least one obstetric outcome. Included studies required the control group to comprise women with untreated endometriosis at the time of pregnancy.

Article abstracts were screened, and all articles meeting the inclusion criteria were read in full. Reference lists were reviewed to identify additional studies for inclusion. Eligibility was firstly assessed based on titles and abstracts. Full manuscripts were then obtained for all appropriate studies. Decision for final inclusion was made (by SM) after detailed reading of the papers in full.

### Bias Assessment

Two authors (SM and MH) independently assessed the risk of bias, using the ROBINS-I framework (16) for non-randomised studies, in the three eligible studies.

### Data Extraction

A standardised data extraction form was used to summarise information on study design, patient characteristics, endometriosis diagnosis and treatment as well as pregnancy outcomes. Relevant subgroup information such as use of ART or details regarding multiple pregnancy, parity or other confounders was also recorded.

## Results

**Figure 1:**
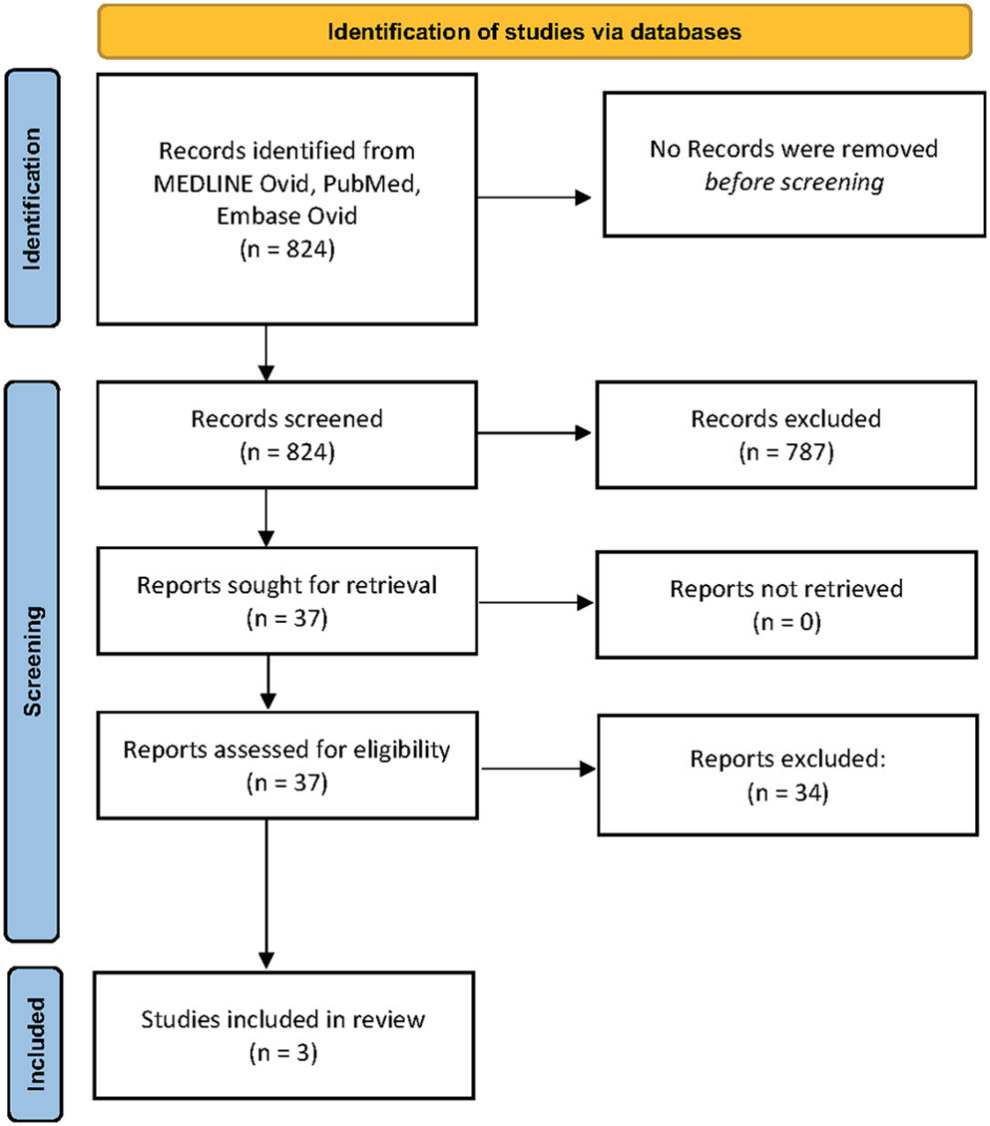
PRISMA flow diagram. From: Page et al. (20).

The electronic database search provided 824 abstracts for screening. Following initial title and abstract screening, 31 articles were reviewed in full to determine suitability for inclusion. A further 6 articles were reviewed in full after reference list review. Three studies (17–19) were included in the final review (Figure 1). A summary of these studies is shown in Table 1.

**Table 1:** Included studies.

**Author(s), (year)**	**Study Design**	**Study Participants**		**Outcome**						
		**Case/Exposed Group**	**Control/Unexposed Group**	**Complication**	**Pre-pregnancy Endometriosis surgery**		**No Endometriosis surgery**		**Crude OR (95% CI)**	***P*** **-value**
Miura et al. (17)	Case-control	Pregnancies (>22weeks gestation) following previous surgical intervention for endometriosis (*n* = 49)	Pregnancies (>22weeks gestation) with a diagnosis of endometriosis based on imaging or symptoms (*n* = 31)	Placenta praevia	With complication	Without complication	With complication	Without complication	2.83 (0.56–14.31)	0.30
					8	41	2	29		
Berlac et al. (19)	Retrospective cohort (population-based)	Births (no gestational limit specified) following surgery for endometriosis (*n* = 3926)	Births (no gestational limit specified) to in patients with endometriosis but no history of endometriosis surgery (*n* = 15405)	PIH PET Abruption PPROM Placenta praevia APH PTB <28 weeks PTB <34 weeks SGA APGAR <7 @5 NND (<28days) Congenital malformation Stillbirth Perineal laceration PPH Instrumental vaginal birth Caesarean	116 130 35 107 136 97 44 231 337 56 23 296 23 314 550 355 1,266	3,810 3,796 3,891 3,819 3,790 3,829 3,882 3,695 3,589 3,870 3,903 3,630 3,903 3,612 3,376 3,571 2,660	288 458 184 342 266 183 137 729 1,023 159 82 1,053 87 218 1,148 1,152 3,340	15,117 14,947 15,221 15,063 15,139 15,222 15,268 14,676 14,382 15,246 15,323 14,352 15,318 15,187 14,257 14,253 12,065	1.60 (1.28–1.99) 1.12 (0.92–1.36) 0.74 (0.52–1.07) 1.23 (0.99–1.54) 2.04 (1.66–2.52) 2.11 (1.64–2.70) 1.26 (0.90–1.78) 1.26 (1.08–1.67) 1.32 (1.16–1.50) 1.39 (1.02–1.89) 1.10 (0.69–1.75) 1.11 (0.97–1.27) 1.04 (0.65–1.65) 6.06 (5.08–7.23) 2.02 (1.82–2.25) 1.23 (1.09–1.39) 1.72 (1.59–1.86)	<0.001 0.29 0.13 0.07 <0.001 <0.001 0.21 0.003 <0.001 0.04 0.77 0.13 0.97 <0.001 <0.001 0.001 <0.001
Thomin et al. (18)^&^	Retrospective cohort	Livebirths following surgery for colorectal DE (*n* = 43)	Livebirths with *in-situ* colorectal DE (*n* = 29)	Caesarean section Difficult extraction Postpartum complications	24 3 10	19 21 33	12 5 9	17 7 20	1.79 (0.69–4.64) 0.20 (0.04–1.06) 0.67 (0.23–1.94)	0.3 0.1 0.6

& *Pre-pregnancy surgical treatment of endometriosis performed, excision or ablation, with “complete” treatment of disease*. *PTB, preterm birth; PP, placenta previa; PET, pre-eclampsia/eclampsia; SGA, small for gestational age; PPROM, premature preterm rupture of membranes; PPH, postpartum haemorrhage; APH, antepartum haemorrhage; DE, deep endometriosis*.

### Study Characteristics

Thomin et al. (18) reported a retrospective cohort study from France, comprising 41 patients with pre-pregnancy colorectal surgery for endometriosis, and 26 women with known *in situ* colorectal endometriosis having no surgical treatment prior to pregnancy. The authors reported on delivery and neonatal outcomes for both groups. All patients were symptomatic of bowel endometriosis (dyschezia, altered bowel habit, pain on defecation, or cyclical rectal bleeding). A clinical diagnosis was made based on visible blue nodules in the posterior fornix, or palpable induration on vaginal and rectal digital examinations, and then confirmed on imaging. All women in the surgical cohort had histologically proven colorectal endometriosis. The non-surgical cohort had *in situ* unresected colorectal endometriosis at the time of pregnancy. The primary outcome of interest was the rate of caesarean section, with secondary outcomes being the incidence of complications dependent on mode of delivery and neonatal outcomes. Fifty percent of cases gave birth by caesarean delivery and rate of caesarean delivery was no different between the groups (OR 1.79; 95% CI 0.69–4.64, *p* = 0.3). Maternal outcome, which included complications during caesarean section or postpartum complications such as endometritis (OR 0.67; 95% CI 0.23–1.94, *p* = 0.6), neonatal outcome [birth weight, a 5 min Apgar score < 7, arterial pH < 7.20, admission to neonatal intensive care unit (NICU) and neonatal death (NND)], and rates of difficulties according to route of delivery (OR 0.20; 95% CI 0.04–1.06, *p* = 0.1) were similar for the two groups. The authors concluded that for women with colorectal endometriosis (*in situ* or previously excised), there was a high rate of caesarean delivery, operative vaginal delivery, and postpartum complications related to delivery. Surgical management of endometriosis pre-pregnancy did not alter these risks.

Miura et al. (17) performed a case control study in Japan, comparing maternal and neonatal outcomes for an endometriosis group (*n* = 80) and controls (*n* = 2,689). They described a subgroup of their endometriosis cohort who had a documented history of surgery for endometriosis (*n* = 49) and separated this group from those with endometriosis who had not undergone pre-pregnancy surgery (*n* = 31). They identified the endometriosis cohort based on laparoscopy with histological confirmation (*n* = 49) or based on imaging findings of endometrioma (*n* = 27) or symptoms (*n* = 4). When comparing the two endometriosis subgroups, those with a history of surgery for endometriosis may have had a greater risk of placenta praevia compared to patients with no prior history of endometriosis surgery but this was not significant, possibly reflecting the small sample size (OR 2.83; 95%CI 0.56–14.31, *p* = 0.30). There was no difference in other maternal outcomes (gestational age, delivery mode, blood loss, hypertensive disorders, gestational diabetes, postpartum haemorrhage (PPH) or placental abruption) or neonatal outcomes (birth weight, Apgar score at 1 and 5 min, umbilical artery pH or NICU admission) between surgery and non-surgery groups.

Berlac et al. (19) conducted a retrospective national cohort study using the Danish Health Register and identified women aged 15–49 years with a diagnosis of endometriosis. The register provides information on diagnoses and interventions from all Danish hospitals. The authors defined “severe endometriosis” as occurring in the patients with endometriosis who had surgical management prior to pregnancy. Pregnancy data was collected from the Danish Medical Birth Register. The diagnosis of endometriosis was confirmed on histological assessment for the surgical group. The authors did not describe how the diagnosis of endometriosis was made in the non-surgical group and no effort was made to define disease severity in this group. The authors reported that almost all pregnancy complications occurred more commonly in the endometriosis group. On comparison of patients with pre-pregnancy surgery for endometriosis (*n* = 3,926) and those with a diagnosis of endometriosis but no surgery (*n* = 15,405), hypertension in pregnancy [OR 1.60 (95% CI 1.28–1.99), *p* < 0.001], placenta praevia [OR 2.04 (1.66–2.52), *p* < 0.001], antepartum haemorrhage (APH) beyond 22 weeks gestation [OR 2.11 (1.64–2.70), *p* < 0.001], PPH [OR 2.02 (1.82–2.25), *p* < 0.001], caesarean section [OR 1.72 (1.59–1.86)], instrumental vaginal birth [OR 1.23 (1.09–1.39), *p* = 0.001], perineal laceration [OR 6.06 (5.08–7.23), *p* < 0.001], PTB <34 weeks gestation [OR 1.26 (1.08–1.67), *p* = 0.002], IUGR [OR 1.32 (1.16–1.50), *p* < 0.001], and 5 min Apgar score <7 [OR 1.39 (1.02–1.89), *p* = 0.04] were increased in women with pre-pregnancy surgery for endometriosis.

### Risk of Bias of Included Studies

Results of the bias assessment are summarised in Supplementary Table 1. All three studies were considered to have critical risk of bias. The three studies had critical risk of bias with no comment regarding completeness of data nor handling of missing data. Moreover, two studies had a serious risk of bias in two further domains, and the third study had a serious risk of bias in three domains, including selection, classification of intervention, and analysis selection. No subgroup analysis adjusted for confounders when comparing patients with endometriosis who had pre-pregnancy surgery with those who did not have surgery.

## Discussion

### The Current Evidence

Whilst the included studies report a potential association between pre-pregnancy endometriosis surgery and several adverse pregnancy outcomes, the risk of bias in each study is critical, impairing the ability to reliably assess the possible role of endometriosis surgery in altering obstetric risk. The true effect of pre-pregnancy surgery for endometriosis may be markedly different from the estimated effect presented by these studies.

#### Control Groups

To assess whether surgery alters the risk of obstetric complications, two control groups should be included: women with known endometriosis without surgery, and women with a previous negative laparoscopy for endometriosis. The study by Miura et al. (17) incorporates these groups but does not directly compare surgical and non-surgically treated endometriosis groups. The sample size is also too small to draw reliable conclusions. Berlac et al. (19) also incorporates these groupings but doesn't directly compare the two endometriosis groups. Moreover, their lack of definition of the non-surgical endometriosis controls makes assessment of the results problematic.

In most published studies examining endometriosis and obstetric risk, the control or “unexposed group” comprised women without a history of a surgical or ultrasound diagnosis of endometriosis (17, 19, 21–35). This is an inappropriate control group to assess if surgery for endometriosis influences pregnancy outcomes. Moreover, the control groups in these studies had no prior laparoscopic assessment to exclude endometriosis, and thus may have had undiagnosed endometriosis (2). Including women with undiagnosed endometriosis in the control groups bias the noted associations towards accepting the null hypothesis.

#### Completeness and Timing of Pre-pregnancy Surgery for Endometriosis

The completeness of surgery and whether residual disease remains at time of conception, could have additional effects. Moreover, the time-period between surgical management and the studied pregnancy has not been specified in any of the reviewed studies. To assess if surgery alters obstetric risk, the surgery needs to pre-date pregnancy, but not be so far ahead to allow recurrence of disease.

In fertility literature, time interval post-surgery appears to be a potentially important variable when assessing the benefit of surgical treatment of endometriosis (36). Thomin et al. (18) describe in detail their surgical technique but not the time between surgery and pregnancy. In contrast, Miura et al. (17) do not describe their surgical technique but do report the surgery to pregnancy interval. An interval of more than 5 years between surgery and pregnancy showed the highest OR for placenta praevia (OR 5.92; 95% CI 1.65–21.30, *p* < 0.01), suggesting that disease recurrence in the intervening years could play a role in obstetric risk.

#### Potential Confounders

The role of ART as a confounder for obstetric risk in women with endometriosis has not been adequately explored and thus the independent effect of endometriosis from that of ART is unknown. Most studies include ART pregnancies which are well-recognised to be associated with an increased risk of poor obstetric outcomes (37), and thus could contribute to bias. Importantly, Farella et al. (38) demonstrated an independent association between ART and PTB, IUGR, and placenta praevia.

The proportion of pregnancies conceived using ART were 19% (*n* = 3,619) for Berlac et al. (19), 40% (*n* = 29) for Thomin (18) and 29% (*n* = 23) for Miura et al. (17) when focusing on women with endometriosis. Miura et al. adjusted for the effect of ART use and found it acted as an independent risk factor for placenta praevia (aOR 2.71; 95% CI 1.70–4.31). There was insufficient information reported about the use of ART between the subgroups of women with pre-pregnancy surgery for endometriosis and women without a history of endometriosis surgery from which to draw conclusions regarding its impact as a confounder.

From the available literature, it is impossible to ascertain whether more severe forms of endometriosis or differing types of endometriosis have an influence on pregnancy outcome (27). Berlac et al. (19) hypothesise that a history of surgery for endometriosis may indicate a more severe form of the disease. The surgical groups described by both Miura et al. (17) and Thomin et al. (18) included patients with moderate or severe endometriosis (Revised American Society for Reproductive Medicine (rASRM) (39) classification grade 3 and 4). In the study by Miura et al. (17), the *in-situ* endometriosis group comprised predominantly cases with endometriomata (based on the author's definitions); it is therefore unknown whether it is the surgical management of endometrioma (or lack of) that could impact on pregnancy outcome. Thomin et al. (18) compared outcomes for those with colorectal endometriosis which affects up to 12% of patients with endometriosis (40). Whilst this is an important group to study, it is not representative of the majority of women with endometriosis and does not examine the role of pre-pregnancy surgical management for women with less severe forms of endometriosis.

A recent meta-analysis (5) concluded there was a higher risk of PTB, placenta praevia, IUGR, and caesarean delivery associated with endometriosis. However, the authors were unable to classify the type or severity of endometriosis, with case groups consisting of any type of endometriosis. In the retrospective cohort study by Uccella et al. (41), the increased risk of placenta praevia was only seen in women with a previous diagnosis of deep endometriosis, but not with ovarian or peritoneal endometriosis in whom risk was similar to controls. Farella et al. (38) prospectively recorded information regarding pre-pregnancy endometriosis type and surgery completed, and broadly described complete surgical treatment of endometriosis. The increased risk of placenta praevia was only noted in women with Rasrm (39) grade 3 or 4 endometriosis, and the absence of endometrioma surgery was associated with an increased risk of IUGR. PTB was associated with prior rectal or bladder surgery for endometriosis. It is theoretically possible that the relationship between endometriosis and obstetric outcome differs depending on the type of endometriosis—deep endometriosis, endometrioma, and superficial disease—and even the location of disease. This relationship has not been adequately investigated; therefore, it is unknown whether any potential effect from surgical treatment will depend on the type and location of endometriosis.

The association between adenomyosis and endometriosis is well-documented (42–45). None of the three included studies presented data regarding the comorbid presence of adenomyosis. Two Japanese cohort studies have demonstrated an association between adenomyosis and several adverse pregnancy outcomes (46, 47). In women with both endometriosis and adenomyosis, even after surgical excision, poorer fertility outcomes are noted compared to patients without adenomyosis (45). It is unknown whether it is adenomyosis, endometriosis, or a combination of both that contributes to obstetric risk. Shi et al. (48) examined the risk of obstetric complications in women with infertility and coexisting endometriosis and adenomyosis, and despite the lack of control group, the high percentages of obstetric complications suggest that adenomyosis is a potential confounder.

### Future Research Needs

Ultimately, prospective studies are required to firstly confirm or refute the possible associations between endometriosis and obstetric complications, and secondly to investigate whether pre-pregnancy management with either medical or surgical modalities alters this risk. At present, the predominantly retrospective literature focuses on heterogenous endometriosis “case” groups made up of women with endometriosis who have undergone surgery, but whether disease was treated, and the completeness of surgery is poorly defined. Future prospective studies should examine whether complete pre-pregnancy surgical treatment of endometriosis alters pregnancy outcomes compared to women without endometriosis, and to women who have untreated endometriosis. Surgery for endometriosis indicated by pain alone, vs. surgery in the setting of infertility and endometriosis may be associated with different obstetric outcomes. Studies should control for the impact of ART as well as controlling for imaging evidence of adenomyosis as a confounder to obstetric outcomes in women following surgical treatment of endometriosis.

Should the link between endometriosis and adverse pregnancy outcome be confirmed by well-designed prospective studies, then a pathophysiological link between these entities also warrants further investigation to identify possible treatment targets and prevention options. Several authors have hypothesised that the alterations at the endometrial-myometrial junctional zone—particularly with spiral artery remodelling—seen in endometriosis may be the link between endometriosis and adverse pregnancy outcomes (12, 49, 50), however this remains highly speculative. Yet, if this is the case, then surgical management of endometriosis would seem unlikely to improve pregnancy outcomes. Endometriosis has also been linked with increased peritoneal cavity inflammation and higher concentrations of cytokines and angiogenic factors (13, 27, 51). It is possible that pre-pregnancy surgical treatment of endometriosis lesions may reduce the presence of these inflammatory substances, though this is not supported by the evidence to date. Cha et al. (52) hypothesise that the obstetric risk noted in women with endometriosis may be due to an inherent predisposition, rather than the direct presence of the lesions. In the current literature, the most commonly cited adverse pregnancy outcome linked with endometriosis is placenta praevia (23). Authors hypothesise that this could be due to altered endometrial receptivity, endometrial inflammation, inadequate uterine contractility, and alteration in the endometrial-myometrial junctional zone (23). The role of surgical treatment in ameliorating these effects pre-pregnancy is unknown.

## Conclusion

There is insufficient evidence available to draw reliable conclusions regarding the role for pre-pregnancy endometriosis surgery in altering the risk of adverse obstetric outcomes. The available studies comparing patients with pre-pregnancy surgery for endometriosis with patients with *in-situ* endometriosis report no improvement in pregnancy outcomes following surgery. Indeed, pre-pregnancy surgery for endometriosis may be associated with worse obstetric outcomes including increased risks of placenta praevia, caesarean delivery, obstetric haemorrhage, gestational hypertensive conditions, PTB and SGA, though due to study bias the true effect is unknown.

It remains unknown whether the phenotype of endometriosis (superficial, ovarian, or deep) or the extent of surgical treatment is important. Moreover, given ART is more commonly utilised in women with endometriosis than those without, it is possible that the presence of endometriosis in addition to the need for ART to conceive may confer even higher obstetric risks. Prospective studies are required to examine the role of surgical therapies for endometriosis and the effect on pregnancy outcomes. They must also assess the impact of comorbid adenomyosis and mode of conception, as well as investigate the biological theories linking endometriosis and adverse pregnancy outcomes.

## Author Contributions

SM: literature search, data extraction, study selection and literature review, and authorship of manuscript. PR, CS, and VR: authorship of manuscript. MH: guidance regarding concept and authorship of manuscript. All authors contributed to the article and approved the submitted version.

## Conflict of Interest

The authors declare that the research was conducted in the absence of any commercial or financial relationships that could be construed as a potential conflict of interest.

## Publisher's Note

All claims expressed in this article are solely those of the authors and do not necessarily represent those of their affiliated organizations, or those of the publisher, the editors and the reviewers. Any product that may be evaluated in this article, or claim that may be made by its manufacturer, is not guaranteed or endorsed by the publisher.

## References

[1] Vercellini P Viganò P Somigliana E Fedele L. Endometriosis: pathogenesis and treatment. Nat Rev Endocrinol. (2014) 10:261–75. 10.1038/nrendo.2013.255 24366116

[2] Rowlands IJ Abbott JA Montgomery GW Hockey R Rogers P Mishra GD. Prevalence and incidence of endometriosis in Australian women: a data linkage cohort study. BJOG. (2021) 128:657–65. 10.1111/1471-0528.16447 32757329

[3] Borisova AV Konnon SRD Tosto V Gerli S Radzinsky VE. Obstetrical complications and outcome in patients with endometriosis. J Matern Fetal Neonatal Med. (2020) 33:1–15. 10.1080/14767058.2020.1793326 32674641

[4] Lalani S Choudhry AJ Firth B Bacal V Walker M Wen SW. Endometriosis and adverse maternal, fetal and neonatal outcomes, a systematic review and meta-analysis. Hum Reprod. (2018) 33:1854–65. 10.1093/humrep/dey269 30239732 PMC6145420

[5] Zullo F Spagnolo E Saccone G Acunzo M Xodo S Ceccaroni M. Endometriosis and obstetrics complications: a systematic review and meta-analysis. Fertil Steril. (2017) 108:667–72.e5. 10.1016/j.fertnstert.2017.07.019 28874260

[6] Kobayashi H Kawahara N Ogawa K Yoshimoto C. A Relationship between endometriosis and obstetric complications. Reprod Sci. (2020) 27:771–8. 10.1007/s43032-019-00118-0 32046459

[7] Fernando S Breheny S Jaques AM Halliday JL Baker G Healy D. Preterm birth, ovarian endometriomata, and assisted reproduction technologies. Fertil Steril. (2009) 91:323–30. 10.1016/j.fertnstert.2008.01.096 18384780

[8] Stephansson O Kieler H Granath F Falconer H. Endometriosis, assisted reproduction technology, and risk of adverse pregnancy outcome. Hum Reprod. (2009) 24:2341–7. 10.1093/humrep/dep186 19439428

[9] Vallvé-Juanico J Houshdaran S Giudice LC. The endometrial immune environment of women with endometriosis. Hum Reprod Update. (2019) 25:564–91. 10.1093/humupd/dmz018 31424502 PMC6737540

[10] Filby CE Rombauts L Montgomery GW Giudice LC Gargett CE. Cellular origins of endometriosis: towards novel diagnostics and therapeutics. Semin Reprod Med. (2020) 38:201–15. 10.1055/s-0040-1713429 33176364

[11] Grandi G Mueller MD Papadia A Kocbek V Bersinger NA Petraglia F. Inflammation influences steroid hormone receptors targeted by progestins in endometrial stromal cells from women with endometriosis. J Reprod Immunol. (2016) 117:30–8. 10.1016/j.jri.2016.06.004 27371899

[12] Brosens I Pijnenborg R Benagiano G. Defective myometrial spiral artery remodelling as a cause of major obstetrical syndromes in endometriosis and adenomyosis. Placenta. (2013) 34:100–5. 10.1016/j.placenta.2012.11.017 23232321

[13] Bedaiwy MA Dahoud W Skomorovska-Prokvolit Y Yi L Liu JH Falcone T. Abundance and localization of progesterone receptor isoforms in endometrium in women with and without endometriosis and in peritoneal and ovarian endometriotic implants. Reprod Sci. (2015) 22:1153–61. 10.1177/1933719115585145 26037298 PMC5933169

[14] Kobayashi H Kawahara N Ogawa K Yoshimoto C. Shared molecular features linking endometriosis and obstetric complications. Reprod Sci. (2020) 27:1089–96. 10.1007/s43032-019-00119-z 32046439

[15] Lier MCI Malik RF Ket JCF Lambalk CB Brosens IA Mijatovic V. Spontaneous hemoperitoneum in pregnancy (SHiP) and endometriosis - a systematic review of the recent literature. Eur J Obstet Gynecol Reprod Biol. (2017) 219:57–65. 10.1016/j.ejogrb.2017.10.012 29054042

[16] Sterne JA Hernán MA Reeves BC Savović J Berkman ND Viswanathan M. ROBINS-I: a tool for assessing risk of bias in non-randomised studies of interventions. BMJ. (2016) 355:i4919. 10.1136/bmj.i4919 27733354 PMC5062054

[17] Miura M Ushida T Imai K Wang J Moriyama Y Nakano-Kobayashi T. Adverse effects of endometriosis on pregnancy: a case-control study. BMC Pregn Childbirth. (2019) 19:373. 10.1186/s12884-019-2514-1 31640604 PMC6805464

[18] Thomin A Belghiti J David C Marty O Bornes M Ballester M. Maternal and neonatal outcomes in women with colorectal endometriosis. BJOG. (2018) 125:711–8. 10.1111/1471-0528.14221 27428865

[19] Berlac JF Hartwell D Skovlund CW Langhoff-Roos J Lidegaard Ø. Endometriosis increases the risk of obstetrical and neonatal complications. Acta Obstet Gynecol Scand. (2017) 96:751–60. 10.1111/aogs.13111 28181672

[20] Page MJ McKenzie JE Bossuyt PM Boutron I Hoffmann TC Mulrow CD. The PRISMA 2020 statement: an updated guideline for reporting systematic reviews. BMJ. (2021) 372:n71. 10.1136/bmj.n71 33782057 PMC8005924

[21] Porpora MG Tomao F Ticino A Piacenti I Scaramuzzino S Simonetti S. Endometriosis and pregnancy: a single institution experience. Int J Environ Res Public Health. (2020) 17:401. 10.3390/ijerph17020401 31936225 PMC7014217

[22] Nirgianakis K Gasparri ML Radan AP Villiger A McKinnon B Mosimann B. Obstetric complications after laparoscopic excision of posterior deep infiltrating endometriosis: a case-control study. Fertil Steril. (2018) 110:459–66. 10.1016/j.fertnstert.2018.04.036 30098698

[23] Chen I Lalani S Xie RH Shen M Singh SS Wen SW. Association between surgically diagnosed endometriosis and adverse pregnancy outcomes. Fertil Steril. (2018) 109:142–7. 10.1016/j.fertnstert.2017.09.028 29198848

[24] Li H Zhu HL Chang XH Li Y Wang Y Guan J. Effects of previous laparoscopic surgical diagnosis of endometriosis on pregnancy outcomes. Chin Med J. (2017) 130:428–33. 10.4103/0366-6999.199840 28218216 PMC5324379

[25] Glavind MT Forman A Arendt LH Nielsen K Henriksen TB. Endometriosis and pregnancy complications: a Danish cohort study. Fertil Steril. (2017) 107:160–6. 10.1016/j.fertnstert.2016.09.020 27743699

[26] Pan ML Chen LR Tsao HM Chen KH. Risk of gestational hypertension-preeclampsia in women with preceding endometriosis: a nationwide population-based study. PLoS ONE. (2017) 12:e0181261. 10.1371/journal.pone.0181261 28715497 PMC5513453

[27] Saraswat L Ayansina DT Cooper KG Bhattacharya S Miligkos D Horne AW. Pregnancy outcomes in women with endometriosis: a national record linkage study. BJOG. (2017) 124:444–52. 10.1111/1471-0528.13920 26887349

[28] Mannini L Sorbi F Noci I Ghizzoni V Perelli F Di Tommaso M. New adverse obstetrics outcomes associated with endometriosis: a retrospective cohort study. Arch Gynecol Obstet. (2017) 295:141–51. 10.1007/s00404-016-4222-7 27770245

[29] Jacques M Freour T Barriere P Ploteau S. Adverse pregnancy and neo-natal outcomes after assisted reproductive treatment in patients with pelvic endometriosis: a case-control study. Reprod Biomed Online. (2016) 32:626–34. 10.1016/j.rbmo.2016.03.005 27068240

[30] Benaglia L Candotti G Papaleo E Pagliardini L Leonardi M Reschini M. Pregnancy outcome in women with endometriosis achieving pregnancy with IVF. Hum Reprod. (2016) 31:2730–6. 10.1093/humrep/dew210 27664955

[31] Fujii T Wada-Hiraike O Nagamatsu T Harada M Hirata T Koga K. Assisted reproductive technology pregnancy complications are significantly associated with endometriosis severity before conception: a retrospective cohort study. Reprod Biol Endocrinol. (2016) 14:73. 10.1186/s12958-016-0209-2 27809920 PMC5094074

[32] Conti N Cevenini G Vannuccini S Orlandini C Valensise H Gervasi MT. Women with endometriosis at first pregnancy have an increased risk of adverse obstetric outcome. J Matern Fetal Neonatal Med. (2015) 28:1795–8. 10.3109/14767058.2014.968843 25262994

[33] Baggio S Pomini P Zecchin A Garzon S Bonin C Santi L. Delivery and pregnancy outcome in women with bowel resection for deep endometriosis: a retrospective cohort study. Gynecol Surg. (2015) 12:279–85. 10.1007/s10397-015-0901-9

[34] Lin H Leng JH Liu JT Lang JH. Obstetric outcomes in Chinese women with endometriosis: a retrospective cohort study. Chin Med J. (2015) 128:455–8. 10.4103/0366-6999.151077 25673445 PMC4836246

[35] Stern JE Luke B Tobias M Gopal D Hornstein MD Diop H. Adverse pregnancy and birth outcomes associated with underlying diagnosis with and without assisted reproductive technology treatment. Fertil Steril. (2015) 103:1438–45. 10.1016/j.fertnstert.2015.02.027 25813277 PMC4465778

[36] Coccia ME Rizzello F Mariani G Bulletti C Palagiano A Scarselli G. Impact of endometriosis on *in vitro* fertilization and embryo transfer cycles in young women: a stage-dependent interference. Acta Obstetr Gynecol Scand. (2011) 90:1232–8. 10.1111/j.1600-0412.2011.01247.x 21793811

[37] Qin JB Sheng XQ Wu D Gao SY You YP Yang TB. Worldwide prevalence of adverse pregnancy outcomes among singleton pregnancies after *in vitro* fertilization/intracytoplasmic sperm injection: a systematic review and meta-analysis. Arch Gynecol Obstet. (2017) 295:285–301. 10.1007/s00404-016-4250-3 27896474

[38] Farella M Chanavaz-Lacheray I Verspick E Merlot B Klapczynski C Hennetier C. Pregnancy outcomes in women with history of surgery for endometriosis. Fertil Steril. (2020) 113:996–1004. 10.1016/j.fertnstert.2019.12.037 32327240

[39] American Society for Reproductive Medicine. Revised American Society for Reproductive Medicine classification of endometriosis: 1996. Fertil Steril. (1997) 67:817–21. 10.1016/S0015-0282(97)81391-X 9130884

[40] Wills HJ Reid GD Cooper MJ Morgan M. Fertility and pain outcomes following laparoscopic segmental bowel resection for colorectal endometriosis: a review. Aust New Zeal J Obstetr Gynaecol. (2008) 48:292–5. 10.1111/j.1479-828X.2008.00871.x 18532961

[41] Uccella S Manzoni P Cromi A Marconi N Gisone B Miraglia A. Pregnancy after endometriosis: maternal and neonatal outcomes according to the location of the disease. Am J Perinatol. (2019) 36:S91–8. 10.1055/s-0039-1692130 31238367

[42] Vercellini P Consonni D Barbara G Buggio L Frattaruolo MP Somigliana E. Adenomyosis and reproductive performance after surgery for rectovaginal and colorectal endometriosis: a systematic review and meta-analysis. Reprod Biomed Online. (2014) 28:704–13. 10.1016/j.rbmo.2014.02.006 24745831

[43] Leyendecker G Bilgicyildirim A Inacker M Stalf T Huppert P Mall G. Adenomyosis and endometriosis. Re-visiting their association and further insights into the mechanisms of auto-traumatisation An MRI study. Arch Gynecol Obstet. (2015) 291:917–32. 10.1007/s00404-014-3437-8 25241270 PMC4355446

[44] Leyendecker G Wildt L Mall G. The pathophysiology of endometriosis and adenomyosis: tissue injury and repair. Arch Gynecol Obstet. (2009) 280:529–38. 10.1007/s00404-009-1191-0 19644696 PMC2730449

[45] Horton J Sterrenburg M Lane S Maheshwari A Li TC Cheong Y. Reproductive, obstetric, and perinatal outcomes of women with adenomyosis and endometriosis: a systematic review and meta-analysis. Hum Reprod Update. (2019) 25:592–632. 10.1093/humupd/dmz012 31318420

[46] Yamaguchi A Kyozuka H Fujimori K Hosoya M Yasumura S Yokoyama T. Risk of preterm birth, low birthweight and small-for-gestational-age infants in pregnancies with adenomyosis: a cohort study of the Japan Environment and Children's Study. Acta Obstet Gynecol Scand. (2019) 98:359–64. 10.1111/aogs.13498 30367455

[47] Tamura H Kishi H Kitade M Asai-Sato M Tanaka A Murakami T. Complications and outcomes of pregnant women with adenomyosis in Japan. Reprod Med Biol. (2017) 16:330–6. 10.1002/rmb2.12050 29259486 PMC5715891

[48] Shi J Dai Y Zhang J Li X Jia S Leng J. Pregnancy outcomes in women with infertility and coexisting endometriosis and adenomyosis after laparoscopic surgery: a long-term retrospective follow-up study. BMC Pregn Childbirth. (2021) 21:383. 10.1186/s12884-021-03851-0 34006232 PMC8132406

[49] Brosens JJ Pijnenborg R Brosens IA. The myometrial junctional zone spiral arteries in normal and abnormal pregnancies: a review of the literature. Am J Obstet Gynecol. (2002) 187:1416–23. 10.1067/mob.2002.127305 12439541

[50] Exacoustos C Luciano D Corbett B De Felice G Di Feliciantonio M Luciano A. The uterine junctional zone: a 3-dimensional ultrasound study of patients with endometriosis. Am J Obstetr Gynecol. (2013) 209:248.e1–7. 10.1016/j.ajog.2013.06.006 23770466

[51] Bedaiwy MA Falcone T. Peritoneal fluid environment in endometriosis. Clinicopathol Implications Minerva Ginecol. (2003) 55:333–45. 14581858

[52] Cha J Sun X Dey SK. Mechanisms of implantation: strategies for successful pregnancy. Nat Med. (2012) 18:1754–67. 10.1038/nm.3012 23223073 PMC6322836

