# Supplementary material for: Obstetric Outcome After Surgical Treatment of Endometriosis: A Review of the Literature
Source: Front Reprod Health. 2021 Dec 24;3:750750. doi: 10.3389/frph.2021.750750 (PMC9580797; doi:10.3389/frph.2021.750750)
Supplement: Supplementary file 1 [file Table_1.docx]

Supplementary Table S1: Bias Assessment, ROBINS-I Framework

| Author | Year | Bias due to confounding | Bias in selection of participants | Bias in classification of interventions | Bias due to deviations from intended interventions | Bias due to missing | Bias in measurement of outcomes | Bias in selection of the reported result | Overall bias |
| --- | --- | --- | --- | --- | --- | --- | --- | --- | --- |
| Miura | 2019 | Moderate | Serious | Serious | Low | Critical | Low | Serious | Critical |
| Berlac | 2017 | Moderate | Serious | Moderate | Low | Critical | Low | Serious | Critical |
| Thomin | 2016 | Moderate | Moderate | Serious | Low | Critical | Low | Serious | Critical |
